# Six Commonly Used Postoperative Radiographic Alignment Parameters Do Not Predict Clinical Outcome Scores after Unrestricted Caliper-Verified Kinematically Aligned TKA

**DOI:** 10.3390/jpm12091468

**Published:** 2022-09-07

**Authors:** Anand Dhaliwal, Tomas Zamora, Alexander J. Nedopil, Stephen M. Howell, Maury L. Hull

**Affiliations:** 1 College of Medicine, California Northstate University, Elk Grove, CA 95757, USA; 2 Orthopedic Surgery Department, Pontificia Universidad Catolica de Chile, Santiago 8331150, Chile; 3 Orthopädische Klinik König-Ludwig-Haus, Lehrstuhl für Orthopädie der Universität Würzburg, 97074 Würzburg, Germany; 4 Department of Biomedical Engineering, University of California, Davis, CA 95616, USA; 5 Department of Mechanical Engineering, University of California, Davis, CA 95616, USA; 6 Department of Orthopedic Surgery, University of California Davis Medical Center, Sacramento, CA 95817, USA

**Keywords:** total knee arthroplasty, kinematic alignment, reoperation, revision, phenotype

## Abstract

Background: Unrestricted caliper-verified kinematically aligned (KA) TKA restores patient’s prearthritic coronal and sagittal alignments, which have a wide range containing outliers that concern the surgeon practicing mechanical alignment (MA). Therefore, knowing which radiographic parameters are associated with dissatisfaction could help a surgeon decide whether to rely on them as criteria for revising an unhappy patient with a primary KA TKA using MA principles. Hence, we determined whether the femoral mechanical angle (FMA), hip–knee–ankle angle (HKAA), tibial mechanical angle (TMA), tibial slope angle (TSA), and the indicators of patellofemoral tracking, including patella tilt angle (PTA) and the lateral undercoverage of the trochlear resection (LUCTR), are associated with clinical outcome scores. Methods: Forty-three patients with a CT scan and skyline radiograph after a KA TKA with PCL retention and medial stabilized design were analyzed. Linear regression determined the strength of the association between the FMA, HKA angle, PTS, PTA, and LUCTR and the forgotten joint score (FJS), Oxford knee score (OKS), and KOOS Jr score obtained at a mean of 23 months. Results: There was no correlation between the FMA (range 2° varus to −10° valgus), HKAA (range 10° varus to −9° valgus), TMA (range 10° varus to −0° valgus), TSA (range 14° posterior to −4° anterior), PTA (range, −10° medial to 14° lateral), and the LUCTR resection (range 2 to 9 mm) and the FJS (median 83), the OKS (median 44), and the KOOS Jr (median 85) (r = 0.000 to 0.079). Conclusions: Surgeons should be cautious about using postoperative FMA, HKAA, TMA, TSA, PTA, and LUCTR values within the present study’s reported ranges to explain success and dissatisfaction after KA TKA.

## 1. Introduction

**Figure 1:**
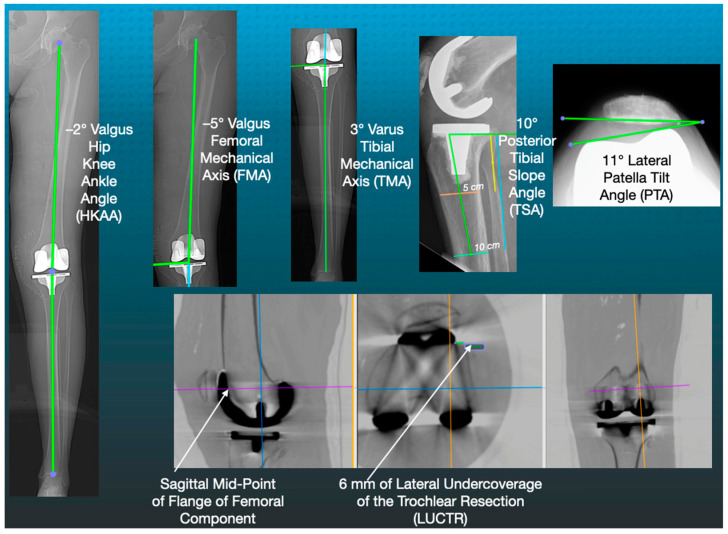
Images of a patient’s left lower limb show the landmarks for measuring the six commonly used radiographic parameters, which were −2° valgus for the HKAA, −5° valgus for the FMA, 3° varus for the TMA, 10° for the TSA, 11° lateral tilt for the PTA, and 6 mm of lateral undercoverage of the trochlear resection at the sagittal midpoint of the flange of the femoral component.

Unrestricted caliper-verified kinematic alignment (KA) total knee arthroplasty (TKA) restores the patient’s prearthritic coronal and sagittal joint lines and Q-angle without the release of intact ligaments, including the posterior cruciate ligament (PCL) [1,2,3]. However, the postoperative consequence is that the femoral mechanical angle (FMA), hip–knee–ankle angle (HKAA), and tibial mechanical angle (TMA) can lie within the varus and valgus outlier categories according to mechanical alignment (MA) criteria (Figure 1) [4]. Therefore, knowing which radiographic parameters are associated with dissatisfaction measured by patient-reported outcome scores (PROMS) could help a surgeon decide whether to rely on them to revise an unhappy patient with a primary KA TKA using MA principles.

In addition to the angles mentioned above, other radiographic parameters are of interest. Several studies reported inconsistent findings concerning the role of the lateral undercoverage of the trochlear resection and the patella tilt angle (PTA) on the risk of reoperation and patient dissatisfaction after KA TKA. For example, one study reported that patients with a valgus femoral and limb phenotype (i.e., high Q-angle) had a small risk of reoperation for anterior knee pain or patella tracking issues (3 of 198 KA TKAs) [5]. Another study confirmed the patella tracking concern about the valgus femoral phenotype because they had more lateral undercoverage of the trochlear resection by the prosthetic trochlea than the varus femoral phenotype, which decreased the Q-angle and increased the risk for lateral patellar tilt as measured by the patella tilt angle (PTA) [6]. However, a study of KA and MA TKA reported similar outcomes, although the incidence of lateral patellar tilt in the KA group was significantly higher (12 of 93) than in the MA group (1 of 93) [7]. Therefore, the role of the lateral undercoverage of the trochlear resection and the PTA on clinical outcomes remains unclear.

Of equal interest is the role that the tibial slope angle (TSA) of the KA tibial baseplate plays when the insert design retains the posterior cruciate ligament (PCL) [1]. In contrast to the MA recommendation of setting the posterior slope within 5–7°, KA matches the slope of the patient’s medial prearthritic tibial slope, which has a range of 1° anterior to 19° posterior in the nonosteoarthritic knee [8]. A TSA greater than the prearthritic knee is associated with tibial component failure from posterior subsidence or insert wear [2]. However, the relationship between the TSA and patient-reported outcome measures is unknown.

Accordingly, the present study determined whether six commonly used postoperative radiographic alignment parameters (Figure 1) are associated with patient-reported clinical outcome scores.

## 2. Materials and Methods

**Figure 2:**
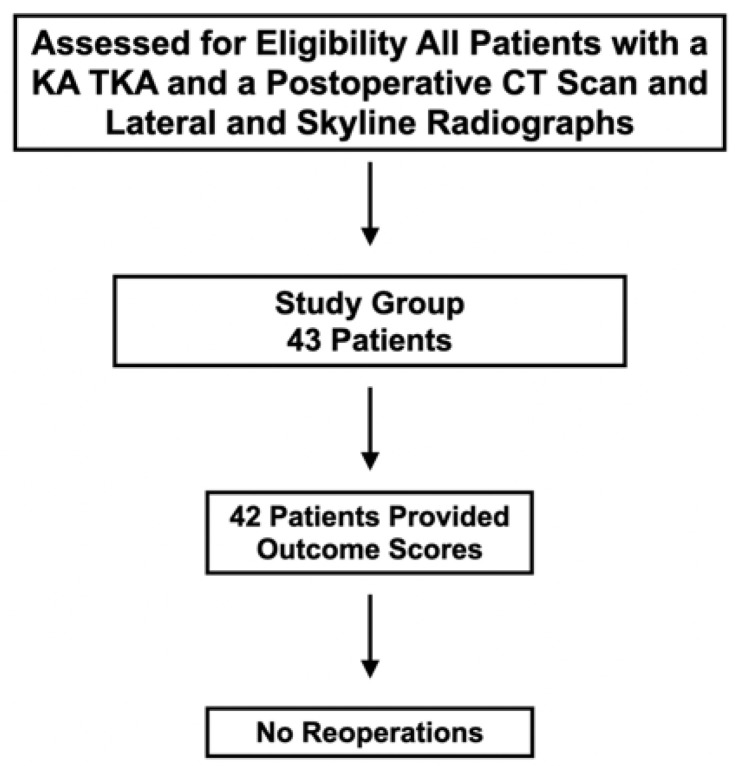
The flowchart shows the number of patients assessed for eligibility included in the study group that provided outcome scores and treated with reoperation.

After obtaining approval from an institutional review board (Pro00063524), an analysis of the senior author’s prospectively acquired radiographic database between July 2020 to December 2021 identified 43 patients that met the following criteria (Figure 2).

**Figure 3:**
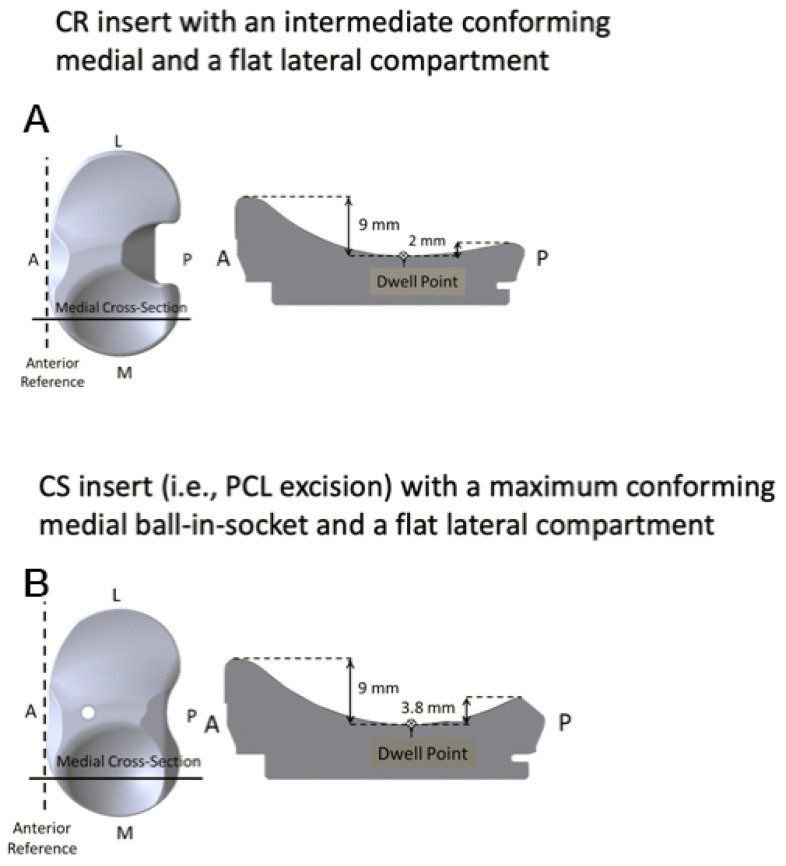
Images show (**A**) the medial sagittal dimensions of a CR (PCL retaining) insert used in the present study) with intermediate conforming tibial articulation and (**B**) for comparison, a CS (PCL substituting) insert with a ball-in-socket tibial articulation.

Included were patients that underwent an unrestricted caliper-verified KA with PCL retention using a medial intermediate conforming and lateral flat articular CR insert design (GMK Sphere, Medacta International, Castel San Pietro, Switzerland) (Figure 3). One patient was excluded because of foot fracture which confounded the outcome scores.

Each patient had an anteroposterior and lateral rotationally controlled, non-weight-bearing, long-leg CT scanogram and axial CT scan, a lateral radiograph, and Laurin skyline views of the patellofemoral joint. Each patient fulfilled the Centers for Medicare & Medicaid Services guidelines for medical necessity for TKA treatment and had (1) Kellgren–Lawrence Grade III to IV osteoarthritis; (2) any severity of clinical varus or valgus deformity; (3) and any severity of flexion contracture. Excluded were patients with prior fractures of the knee treated with open-reduction internal fixation, inflammatory or septic arthritis, and lower extremity neurologic disorders.

A single surgeon (SMH) performed unrestricted caliper-verified KA TKA with manual instruments through a mid-vastus approach and intraoperatively recorded a series of verification checks using a previously described technique [9]. For the femoral component, the internal-external axial (I-E) and varus-valgus (V-V) rotations and the anterior-posterior (A-P) and proximal-distal (P-D) positions were set coincident with the native distal and posterior joint lines by adjusting the caliper-measured thicknesses of the distal and posterior femoral resections to within 0 ± 0.5 mm of those of the femoral component condyles after compensating for the cartilage wear and kerf of the saw blade. The accuracy of setting the femoral component to the KA target with manual instruments was comparable to or better than reported values for MA using robotic instrumentation [10].

For the tibial component, the knee was balanced by adjusting the P-D position, V-V rotation, and the medial slope of the tibial resection to match the patient’s prearthritic tibial joint line [9]. The V-V angle of the tibial resection was adjusted, working in 1°−2° increments, until there was negligible V-V laxity in maximum extension with the spacer block and trial component. This verification check sets 97% of tibial components within the left-to-right symmetry of the nonosteoarthritic lower limbs [3]. The resection’s tibial slope angle (TSA) was set parallel to the medial joint line by adjusting the plane of an angel wing inserted in the tibial guide with a reported mean difference of 0.7° ± 3.2° [1]. A best-fit technique of the anatomic baseplate set the internal-external orientation with a mean 2° external ± 6° deviation from the flexion-extension plane of the knee [11].

A single observer (TZ) measured the six postoperative radiographic alignment parameters using free image-analysis software (Horos Imaging Software, Horos, v3.3.6, Annapolis, MD, USA) (Figure 1). The FMA was 90° minus the angle between a line tangent to the distal femoral component and a line connecting the centers of the femoral head and knee on the A-P CT scanogram of the limb (+varus, −valgus). The HKAA was the angle measured from the lateral side between a line connecting the centers of the femoral head and knee and a line connecting the centers of the knee and ankle on the A-P CT scanogram of the limb minus 180° (+varus, −valgus). The TMA was the angle between a line tangent to the proximal tibial component and a line connecting the centers of the knee and ankle on the A-P CT scanogram of the limb minus 90° (+varus, −valgus) [3]. The TSA was 90° minus the angle formed by a line tangent to the proximal tibial component and a vertical line connecting the mid-points of two transverse lines placed 5 and 10 cm distal to the tibial joint line on the lateral radiograph of the knee (+posterior, −anterior). The PTA was the angle formed by a line tangent to the anterior border of the femoral condyles and a line tangent to the patella–prosthesis interface on the skyline radiographic view (+lateral, −medial) [7,12]. Finally, the lateral undercoverage of the trochlear resection was measured on a multiplane reconstruction of the axial CT scan that projected the femoral component parallel to the lateral lug on the sagittal and coronal views and parallel to both lugs on the axial view (Figure 1). The undercoverage was the millimeter distance between the lateral edge of the femoral resection and the sagittal midpoint of the flange of the femoral component. 

Between January and May 2022, patients were sent a questionnaire by email and postal service asking them whether the TKA had a reoperation and to complete and return the FJS (100 best and 0 worst), OKS (48 best and 0 worst), and KOOS Jr (100 best and 0 worst).

### Statistical Analysis

Two methods of statistical analysis determined the consistency of measurements for each radiographic parameter. The first method computed the intraclass correlation coefficients (ICCs) to determine the interobserver and intraobserver variability of each radiographic parameter. To quantify interobserver variability, three observers measured images from twelve patients randomly selected from the 43 enrolled in the study. To quantify intraobserver variability, one observer made five measurements on alternating days on five randomly selected TKAs. A two-factor ANOVA with the observer and patient modelled as random effects was performed for each radiographic parameter. Intraobserver and interobserver ICCs were computed using the variance components for observer, patient, and error [13]. The second method determined repeatability (i.e., the precision of measurement), which was quantified as the square root of the pooled variance for a single observer.

Dependent variables were reported as either the mean ± standard deviation (SD) or the median and interquartile range (IQR). Software performed a simple linear regression and computed the correlation coefficient (r-value) and significance of the relationship between the six radiographic parameters and the FJS, OKS, and KOOS Jr scores. Significance was *p* < 0.05.

## 3. Results

The average age of the 43 patients was 69 ± 8 years, and 21 were females. The preoperative clinical characteristics are listed in Table 1.

The ICCs for reproducibility and repeatability were excellent or good for the six commonly used postoperative radiographic parameters (Table 2).

There was a wide range of values for the postoperative radiographic parameters since the components were set to restore the patients’ prearthritic joint lines (Table 3).

None of the six postoperative radiographic parameters correlated significantly with the postoperative patient-reported FJS (median 83 and IQR 50), OKS (median 44 and IQR 11), and KOOS Jr (median 85 and IQR 32) obtained at a mean follow-up of 23 ± 9 months (Table 4).

## 4. Discussion

The most important findings of the present study were that the FMA, HKAA, TMA, PTS, PTA, and LUCTR did not predict the forgotten joint score (FJS), Oxford knee score (OKS), or KOOS Jr score. Hence, measuring these six postoperative radiographic parameters did not discriminate between satisfied and dissatisfied patients after KA TKA.

The lack of an association between these radiographic parameters and satisfaction is consistent and different from other KA and MA TKA studies. One KA TKA study reported a higher incidence of revision in patients with more valgus femoral and limb phenotypes [5]. Some studies of MA TKA report that postoperative alignment does not determine the outcome [14,15], while others report that leaving patients with preoperative varus deformities in mild postoperative varus had better functional outcome scores than those corrected to neutral [16,17]. Therefore, the value of measuring postoperative alignment to predict clinical outcomes remains controversial. 

The KA TKA restoration of native knee medial and lateral tibial compartment forces explains the poor association between alignment and outcome scores [18,19,20,21,22]. Setting the femoral and tibial components coincident to the prearthritic joint lines is critical because minor 1° and 2° varus or valgus and internal-external deviations overload the tibial compartments enough to cause knee stiffness [19,20,23,24]. The reported accuracy of restoring the patient’s prearthritic joint lines with the imageless caliper-verified KA technique with manual instruments is more accurate than robotic instrumentation [10,25]. When a KA TKA patient expresses dissatisfaction, the first check is to determine whether the femoral and tibial components are set correctly by analyzing the thickness of the bone resections measured by a caliper when recorded in the operative note. Each thickness should equal that of the condyle of the femoral component minus 2 mm for cartilage wear and minus 1 mm for the saw blade’s kerf. A second check is to determine whether the coronal alignments of the femoral and tibial components are comparable to the femoral and tibial joint lines on the preoperative radiograph. The deviations should be no more than a few degrees.

The TSA after KA TKA should match the patient’s prearthritic slope, which is different from reported recommended ranges for MA TKA performed with CR and PS inserts. In MA TKA, the TSA range is within 5–7° for the CR design and 0–3° for the PS design. In contrast, the TSA after KA TKA with PCL retention was outside the CR range (i.e., 30% had more than 7° posterior slope and 5% less than 5°) and PS range (i.e., 65% had more than 3° posterior slope and 7% less than 0°) recommended for MA TKA [8]. When a KA TKA patient expresses dissatisfaction, the surgeon should determine the deviation between the preoperative and postoperative TSA. The deviation should be limited to a few degrees, as a tibial component with a slope 5° greater than the prearthritic slope has an increased risk of posterior overload leading to subsidence or insert wear [2].

The PTA’s lack of association with clinical outcomes scores after KA TKA might surprise the MA surgeon because they rotate the femoral component externally relative to the posterior joint line to reduce the PTA and promote patella tracking. However, one case-matched study showed that KA and MA TKA had comparable clinical outcomes, although the KA TKA had a greater incidence and magnitude of lateral patella tilt [7]. Another comparison failed to show a difference in the PTA, as the proportion of KA TKA in the present study within the PTA ranges of 0–3°, 4–7°, and 8° or greater were comparable to MA TKA (i.e., 65% vs. 56%, 21% vs. 28%, and 14% vs. 16%) [26]. Therefore, a PTA within the −3° medial to 11° lateral range of the present study is not an indicator for patella-stabilizing revision surgery.

The 4 ± 1.5 mm (range 2 to 9 mm) lateral undercoverage of the trochlear resection with a femoral component designed for MA in the present study was comparable to another study of KA that reported 3 ± 2.9 mm (range 0 to 6 mm) of undercoverage for a different brand of MA-designed femoral component [6]. Similar findings with different femoral components between these studies suggest that a change in femoral component design explicitly for KA might be a strategy to reduce the risk of undercoverage. However, the benefit of a KA design needs validation since there was no association between the level of undercoverage and clinical outcome scores in the present study. Finally, 2 to 9 mm of undercoverage is not an indicator for revision surgery in a dissatisfied patient.

The study has several limitations. First, other than postoperative alignment, the predictors of dissatisfaction, such as prior knee surgery, preoperative varus or valgus osteoarthritic alignment, BMI, mental health, activity level, age, and sex, were not analyzed. Finally, other studies should confirm or refute these results by analyzing more extensive case series and different femoral component designs.

## 5. Conclusions

Surgeons should be cautious about using postoperative measurements of the coronal, sagittal, and axial alignment of the femoral and tibial components to explain success and dissatisfaction after KA TKA and to indicate revision surgery.

## Figures and Tables

**Table 1:** Preoperative characteristics, knee conditions, and function scores for the 43 patients in the present study.

Preoperative Characteristics	Values ± Standard Deviation (Range)
Age	70 ± 8 years (54 to 84)
Sex	21 females and 22 males
Body mass index	32 ± 7 kg/m^2^ (21 to 52)
Extension	8 ± 7° (0 to 29°)
Flexion	113 ± 6° (100 to 125°)
Type of osteoarthritic knee deformity	67% varus, 26% valgus, and 7% patellofemoral
Radiographic knee deformity (+varus, −valgus)	−1 ± 7 (14 to −17)
Kellgren–Lawrence classification	2% II, 35% III, and 63% IV
Oxford knee score (48 is best and 0 is worst)	21 ± 8 points (6 to 39)
KOOS Jr (100 is best and 0 is worst)	43 ± 14 points (16 to 73)

**Table 2:** The interobserver and intraobserver intraclass correlation coefficients (ICC values), repeatability, and agreement classification for measurements of the six postoperative alignment parameters.

Postoperative Alignment Parameter	Interobserver Intraclass Correlation	Intraobserver Intraclass Correlation	Repeatability
Femoral mechanical angle (FMA)	ICC = 0.92 *	ICC = 0.92 *	0.5°
Tibial mechanical angle (TMA)	ICC = 0.94 *	ICC = 0.94 *	0.5°
Hip–knee–ankle angle (HKAA)	ICC = 0.95 *	ICC = 0.95 *	0.6°
Tibial slope angle (TSA)	ICC = 0.82 ^#^	ICC = 0.83 ^#^	0.8°
Patella tilt angle (PTA)	ICC = 0.88 ^#^	ICC = 0.89 ^#^	0.9°
Lateral undercoverage of the trochlear resection (LUCTR)	ICC = 0.92 *	ICC = 0.93 *	0.5 mm

* Excellent agreement (ICC > 0.9); ^#^ good agreement (ICC 0.75 to 0.9).

**Table 3:** The values, proportion, and type of outlier according to MA criteria for the six postoperative radiographic parameters.

Postoperative Alignment Parameter	Minimum to Maximum Mean ± SD	Varus and Slope MA Outlier Type or Range	Valgus and Slope MA Outlier Type or Range
Femoral mechanical angle (FMA)	2° varus to −9° valgus −3° ± 2.4°	0% varus	53% valgus (<−3°)
Tibial mechanical angle (TMA)	10° varus to −9° valgus 4° ± 2.6°	63% varus (<87°)	0% valgus
Hip–knee–ankle angle (HKAA)	10° varus to −7° valgus 1° ± 3.9°	26% varus (>3°)	14% valgus (<−3°)
Tibial slope angle (TSA)	−4° anterior to 11° posterior 5° ± 3.8°	28% within 0–3°	16% within 0–3°
Patella tilt angle (PTA)	−3° medial to 11° lateral 3° ± 3.1°		
Lateral undercoverage of the trochlear resection (LUCTR)	2 to 9 mm 4 ± 1.5 mm	60% within 3–6 mm	30% > 6 mm

**Table 4:** The correlation coefficient (r) and significance between six postoperative radiographic parameters and the postoperative forgotten joint score, Oxford knee score, and KOOS Jr.

Postoperative Alignment Parameter	Forgotten Joint Score	Oxford Knee Score	KOOS Jr
Femoral mechanical angle (FMA)	r = −0.093 *p* = 0.552	r = −0.107 *p* = 0.494	r = −0.106 *p* = 0.946
Tibial mechanical angle (TMA)	r = 0.138 *p* = 0.376	r = 0.059 *p* = 0.7091	r = 0.265 *p* = 0.086
Hip–knee–ankle angle (HKAA)	r = 0.133 *p* = 0.403	r = 0.082 *p* = 0.608	r = −0.099 *p* = 0.535
Tibial slope angle (TSA)	r = −0.157 *p* = 0.316	r = −0.281 *p* = 0.068	r = −0.205 *p* = 0.187
Patella tilt angle (PTA)	r = 0.107 *p* = 0.498	r = 0.122 *p* = 0.4430	r = 0.214 *p* = 0.173
Lateral undercoverage of the trochlear resection (LUCTR)	r = 0.088 *p* = 0.5739	r = 0.007 *p* = 0.966	r = −0.041 *p* = 0.797

## Data Availability

Not applicable.
